# Comparison of Fecal Collection Methods for Microbiome and Metabolomics Studies

**DOI:** 10.3389/fcimb.2018.00301

**Published:** 2018-08-28

**Authors:** Zheng Wang, Christine P. Zolnik, Yunping Qiu, Mykhaylo Usyk, Tao Wang, Howard D. Strickler, Carmen R. Isasi, Robert C. Kaplan, Irwin J. Kurland, Qibin Qi, Robert D. Burk

**Affiliations:** ^1^Department of Epidemiology and Population Health, Albert Einstein College of Medicine, Bronx, NY, United States; ^2^Department of Pediatrics, Albert Einstein College of Medicine, Bronx, NY, United States; ^3^Department of Biology, Long Island University, Brooklyn, NY, United States; ^4^Department of Medicine, Stable Isotope and Metabolomics Core Facility, Diabetes Center, Albert Einstein College of Medicine, Bronx, NY, United States; ^5^Public Health Sciences Division, Fred Hutchinson Cancer Research Center, Seattle, WA, United States; ^6^Microbiology and Immunology, Albert Einstein College of Medicine, Bronx, NY, United States; ^7^Obstetrics, Gynecology and Women's Health, Albert Einstein College of Medicine, Bronx, NY, United States

**Keywords:** fecal microbiome, metabolomics, sampling methods, multi-omics integration, integrative analysis

## Abstract

**Background:** Integrated microbiome and metabolomics analyses hold the potential to reveal interactions between host and microbiota in relation to disease risks. However, there are few studies evaluating how field methods influence fecal microbiome characterization and metabolomics profiling.

**Methods:** Five fecal collection methods [immediate freezing at −20°C without preservative, OMNIgene GUT, 95% ethanol, RNA*later*, and Flinders Technology Associates (FTA) cards] were used to collect 40 fecal samples from eight healthy volunteers. We performed gut microbiota 16S rRNA sequencing, untargeted metabolomics profiling, and targeted metabolomics focusing on short chained fatty acids (SCFAs). Metrics included α-diversity and β-diversity as well as distributions of predominant phyla. To evaluate the concordance with the “gold standard” immediate freezing, the intraclass correlation coefficients (ICCs) for alternate fecal collection systems were calculated. Correlations between SCFAs and gut microbiota were also examined.

**Results:** The FTA cards had the highest ICCs compared to the immediate freezing method for α-diversity indices (ICCs = 0.96, 0.96, 0.76 for Shannon index, Simpson's Index, Chao-1 Index, respectively), followed by OMNIgene GUT, RNA*later*, and 95% ethanol. High ICCs (all >0.88) were observed for all methods for the β-diversity metric. For untargeted metabolomics, in comparison to immediate freezing which detected 621 metabolites at ≥75% detectability level, 95% ethanol showed the largest overlapping set of metabolites (*n* = 430; 69.2%), followed by FTA cards (*n* = 330; 53.1%) and OMNIgene GUT (*n* = 213; 34.3%). Both OMNIgene GUT (ICCs = 0.82, 0.93, 0.64) and FTA cards (ICCs = 0.87, 0.85, 0.54) had acceptable ICCs for the top three predominant SCFAs (butyric acid, propionic acid and acetic acid). Nominally significant correlations between bacterial genera and SCFAs (*P* < 0.05) were observed in fecal samples collected by different methods. Of note, a high correlation between the genus *Blautia* (known butyrate producer) and butyric acid was observed for both immediate freezing (*r* = 0.83) and FTA cards (*r* = 0.74).

**Conclusions:** Four alternative fecal collection methods are generally comparable with immediate freezing, but there are differences in certain measures of the gut microbiome and fecal metabolome across methods. Choice of method depends on the research interests, simplicity of fecal collection procedures and ease of transportation to the lab, especially for large epidemiological studies.

## Introduction

Over the past decade, the microbiota's potential impact on human chronic diseases has garnered increasing interest. Many health conditions, such as diabetes, cardiovascular diseases, and cancers, were found to be associated with microbiota, particularly with the gut microbiota (Ley, 2010; Kostic et al., 2014; Forslund et al., 2015; Vogtmann and Goedert, 2016; Chong and Xia, 2017). Interactions between host and microbiota occur primarily through evolutionarily conserved chemical dialogs that involve a multitude of metabolites and pathways (Martin et al., 2009; Candela et al., 2011). Thus, integrating analyses of microbiome and related metabolites should facilitate understanding the role of microbiota in human chronic diseases (Fiehn, 2002; Patti et al., 2012; Chong and Xia, 2017).

The measurements of the gut microbiota are influenced by numerous factors, including “wet-lab” protocols used for assaying specimens, and “dry-lab” approaches used in data processing. In addition, the field methods used for fecal sample collection comprise another critical methodological feature that is often difficult or impossible to remediate once a protocol has been fielded (Cardona et al., 2012; Choo et al., 2015; Sinha et al., 2015, 2016). A number of fecal collection methods, such as 95% ethanol, OMINIgene GUT Kit, RNA*later* Stabilization Solution, fecal occult blood test (FOBT) cards, and fecal immunochemical test tubes, have been examined for gut microbiota profiling, and compared to immediate freezing at −20°C, which is considered as the “gold standard” (Nechvatal et al., 2008; Flores et al., 2015; Voigt et al., 2015; Sinha et al., 2016; Song et al., 2016; Vogtmann et al., 2017b). These collection systems showed relatively high reproducibility and stability at ambient temperature. Nevertheless, variations were observed across different methods (Nechvatal et al., 2008; Flores et al., 2015; Voigt et al., 2015; Sinha et al., 2016; Song et al., 2016; Vogtmann et al., 2017b). However, it is unclear if specific collection methods are adequate for integrated microbiome and metabolomics studies. A recent study compared the aforementioned fecal collection methods for untargeted metabolomics profiling, and the 95% ethanol method showed the highest concordance with the “gold standard” (Loftfield et al., 2016). To the best of our knowledge, no studies have compared fecal collection methods for both gut microbiota profiling and fecal metabolomics simultaneously.

In order to determine the optimal collection method for integrated microbiome and metabolomics studies, we compared five fecal collection methods for gut microbiota 16S rRNA gene V4 region sequencing and metabolomics profiling. FTA cards, which make use of a swab sample that is smeared on a card and then allowed to dry, use a nucleic acid stabilizer and are similar in format to FOBT cards (Vandeputte et al., 2017). This supports the rationale for FTA cards as effective tools which may provide comparable microbiota diversity results compared with immediate freezing (Song et al., 2016; Wong et al., 2017), without the need for long term cold storage. In addition to untargeted metabolomics profiling, we also designed a targeted metabolomics panel focusing on short chained fatty acids (SCFAs), which are major metabolites produced by gut microbiota. This part of the study entailed measurements, across different fecal collection methods, of fecal SCFA concentrations as well as correlations between SCFAs and gut microbiota.

## Materials and methods

### Fecal specimen collection

Five different methods (OMNIgene GUT (DNA Genotek, Ottawa, Canada), 95% ethanol, RNA*later* (Invitrogen, Carlsbad, CA), FTA cards (GE Healthcare, Chicago, IL), and immediate freezing at −20°C were used to collect 40 fecal samples from eight healthy volunteers. The fecal samples were self-collected using a disposable paper inverted hat (Protocult collection device, ABC Medical Enterprises, Inc., Rochester, MN). Each participant collected five fecal samples in total from a single specimen, including one sample for each of the five different methods. The participants sampled the stool with a plastic applicator, spreading a small amount on a Whatman FTA card, as the first sample. They collected a second sample which was placed in a supplied container including a stabilizer (RNA*later*) and 0.5 mm diameter glass beads and instructed to shake the tube in order to mix the stool and the preservative which stabilizes DNA and RNA (Flores et al., 2012). The third sample was placed in the OMNIgene GUT tube, using the supplied applicator and following the manufacturer's directions. The fourth sample was placed in a supplied tube containing 95% ethanol and glass beads. All samples collected by the aforementioned four methods were left at room temperature. The fifth sample was placed in a supplied container with no solution, placed in a plastic bag and put into a household freezer immediately. Within 24 h, all samples were delivered to the laboratory using a styrofoam box containing ice packs. Upon arrival at the lab, two aliquots of each fecal sample were immediately created for the gut microbiome and metabolomics analyses and stored at −80°C.

The study was reviewed and approved by the institutional review board (IRB) at the Albert Einstein College of Medicine. Written informed consent was obtained from all participants.

### DNA extraction and 16S rRNA gene V4 region sequencing

Laboratory procedures were conducted under a hood (AirClean Systems, Creedmoor, NC) to limit environmental contamination. Total DNA was extracted from stool samples with the PowerLyzer PowerSoil DNA Isolation Kit (MO BIO laboratories Inc., Carlsbad, CA), following the manufacturer's procedures. Briefly, a 100 μl homogenized aliquot from the RNA*later*, 95% ethanol and OMNIgene GUT tube samples, a small scoopful of the immediate freezing sample and 1/4 of the FTA card spot were each added to PowerLyzer bead tubes with 60 μl of Solution C1 (vortexed to mix) and beaten with a FastPrep-24 homogenizer (MP Biomedicals, Santa Ana, CA) at speed 6.0 for 40 s. The samples were centrifuged at 10,000 *g* for 30 s and the supernatant was removed and placed in a new collection tube. The DNA was isolated by column purification and collected in 100 μl of elution buffer (Solution C6).

PCR amplification of the V4 hypervariable region of the 16S rRNA gene was performed using primers 16SV4_515F and 16SV4_806R (Caporaso et al., 2012) each with 12-bp unique Golay barcodes, resulting in unique dual barcodes for each forward and reverse primer pair. PCR reactions were performed with 16.25 μl of nuclease-free PCR-grade water, 2.5 μl of 10X Buffer w/MgCl_2_ (Affymetrix,Santa Clara, CA), 1 μl of MgCl_2_ (25 mM, Affymetrix), 0.5 μl of dNTPs (10 mM, Roche, Pleasanton, CA), 0.25 μl of AmpliTaq Gold DNA Polymerase (5 U/μl, Applied Biostystems, Foster City, CA), 0.5 μl of HotStart-IT FideliTaq (2.5 U/μl, Affymetrix), 1 μl of each primer (5 μM), and 2 μl of extracted DNA. Thermal cycling conditions included an initial denaturation at 95°C for 5 min; followed by 15 cycles at 95°C for 1 min, 55°C for 1 min, and 68°C for 1 min; followed by 15 cycles at 95°C for 1 min, 60°C for 1 min, and 68°C for 1 min; and a final extension for 10 min at 68°C on a GeneAmp PCR System 9700 (Applied Biosystems).

Dual indexed PCR products were isolated and combined and 100 μl of the pooled products were run on a 4% agarose gel at 80 V for 2 h. The bands (~450 bp) were excised from the agarose gel and purified using a QIAquick Gel Extraction Kit (QIAGEN, Valencia, CA) and eluted in 30 μl of elution buffer. The purified PCR products were quantified using a Qubit 2.0 Fluorometic High Sensitivity dsDNA Assay (Life Technologies, Carlsbad, CA).

A sequencing library was prepared using KAPA LTP Library Preparation Kit (Roche Sequencing Solutions, Pleasanton, CA) according to the manufacture's protocol. The size integrity of the amplicon was validated with a 2100 Bioanalyzer (Agilent Technologies, Santa Clara, CA) at the Albert Einstein College of Medicine Genomics Core. High-throughput amplicon sequencing was conducted on a MiSeq (Illumina, San Diego, CA) using 2 × 300 paired-end fragment reads at the Albert Einstein College of Medicine Sequencing Core.

### Microbiome bioinformatics analysis

Illumina reads were quality trimmed to remove bases with PHRED quality scores below 25 using prinseq version 0.20.4 (Schmieder and Edwards, 2011). Samples were then demultiplexed based on the sample specific dual Golay barcode combinations. To generate a full length 16S rRNA V4 region sequence, the paired-end reads were joined into a single sequence with the FLASH algorithm (Magoc and Salzberg, 2011). After quality control, one sample was excluded in the analysis due to unexpected low reads (<1,000). The average coverage was ~ 18,000 reads per sample.

Microbiome bioinformatics analysis was performed using the Quantitative Insights Into Microbial Ecology (QIIME) software package, version 1.9 (Caporaso et al., 2010b). Sequences were clustered into operational taxonomic units (OTUs) based on ≥97% similarity by the UCLUST algorithm. Phylogenetic reconstruction was performed by first aligning OTU representative sequences using PyNAST (Caporaso et al., 2010a). Taxonomy was then assigned using the QIIME closed reference OTU picking method with the Greengenes database, version 13.5 (DeSantis et al., 2006; McDonald et al., 2012). We calculated the relative abundance from phylum level to genus level for each collection method. α-diversity indices (Shannon index, Simpson's Index, and Chao 1 index) and β- diversity Bray-Curtis distances were calculated using the R phyloseq/vegan package (McMurdie and Holmes, 2013; Oksanen, 2015).

### Metabolomics profiling

Sample extraction and derivatization for untargeted profiling: Metabolites extraction was performed with methanol (ethanol for ethanol collected samples): water = 3:1. The solutions in OMINIgene GUT kit and RNA*later* were considered as water. After vortex and sonication for 5 min, the samples were centrifuged at 15,000 rpm for 15 min. A volume of 500 μl of supernatant was added with internal standards (10 ul of 10 nmol U^13^C citrate and 5 nmol of U^13^C succinate) and dried under gentle nitrogen flow. Samples collected with RNA*later* were not dried after the overnight drying, and were not used for the next step.

Dried samples from ethanol, immediate freezing, FTA cards, and Omni kit were subjected to a two-step derivatization (methoximation and silylation) as described previously (Qiu et al., 2009). The samples were analyzed by gas chromatography mass spectrometry (GC-MS, Agilent, USA) with a 30-meter DB-5MS column. The oven program was initiated with 60°C for 1 min, and increased to 320°C and kept for 5 min. A full scan mode was used with the mass range of 35–600 Da. Raw data was analyzed in Genedata Expressionist (Genedata, Basel, Switzerland) software. Metabolite annotation was performed by comparing the mass spectrum and retention time to our in-house libraries and commercially available libraries (i.e., Fiehn and NIST).

Targeted short chain fatty acids analysis: Feces samples collected with 4 methods (immediate freezing, OMINIgene GUT, RNA*later*, and FTA cards) were used for SCFA analysis with propyl chloroformate (PCF) derivatization. Due to interference of ethanol with propanol, samples collected with ethanol were not used for PCF analysis (ethanol will react with SCFAs to form ethyl ester under this derivatization condition).

A volume of 1 mL water was added to dry samples (immediate freezing and FTA cards). All samples were vortexed for 2 min, and sonicated for 5 min. The supernatant was collected after centrifugation of 15 min at 15,000 rpm. A volume of 500 μl of supernatant was transferred into a new glass tube for derivatization with 10 μl of internal standards (500 ug/ml propanoic acid_D5, and 100 μg/mL butyric acid_D7). The derivatization and GC-MS analysis followed our previous protocol (Zheng et al., 2013). Chemstation was used for data analysis.

### Statistical analysis

Kruskal-Wallis test was applied to compare the differences in the microbial α-diversity indices (Shannon index, Simpson index and Chao-1 index) across five fecal collection methods. PERMANOVA with Bray-Curtis dissimilarity and principal-coordinate analysis (PCoA) were carried out for the microbial β-diversity analyses. To evaluate the concordance of different fecal collection methods compared with the gold standard immediate freezing, we calculated the intraclass correlation coefficients (ICCs) for the three α-diversity indices, the β-diversity metric Bray-Curtis distances, and the relative abundances of the top three dominant phyla (Actinobacteria, Bacteroidetes, Firmicutes). We calculated the distance-based ICC for β-diversity and the 95% CIs using 1,000 bootstrap value according to the algorithm described elsewhere (Vogtmann et al., 2017a). Square-root transformation was conducted for the relative abundance of taxonomic units before analysis (Nakatsu et al., 2015). For untargeted metabolomics, metabolite values were first normalized using Quantile normalization (Bolstad et al., 2003) for each method and then Log10 transformation was performed before analyses. Missing values were imputed with ½ minimum values for a given metabolite within one method. ICCs between collection methods for metabolites with ≥75% detectability (e.g., measured in ≥6 out of 8 participants) were calculated. For targeted metabolomics, ICCs between collection methods for eight SCFAs with ≥75% detectability were calculated. In addition, Spearman correlations between microbial genera and SCFAs were computed within each method. R packages vegan, icc, DESeq2, and phyloseq were used for the statistical analyses (Anders and Huber, 2010; McMurdie and Holmes, 2013; Love et al., 2014; Oksanen, 2015).

### Accession number

The sequencing data are available at the Sequence Read Archive (SRA) under accession number SRP153121.

## Results

### Microbiome analyses

**Figure 1:**
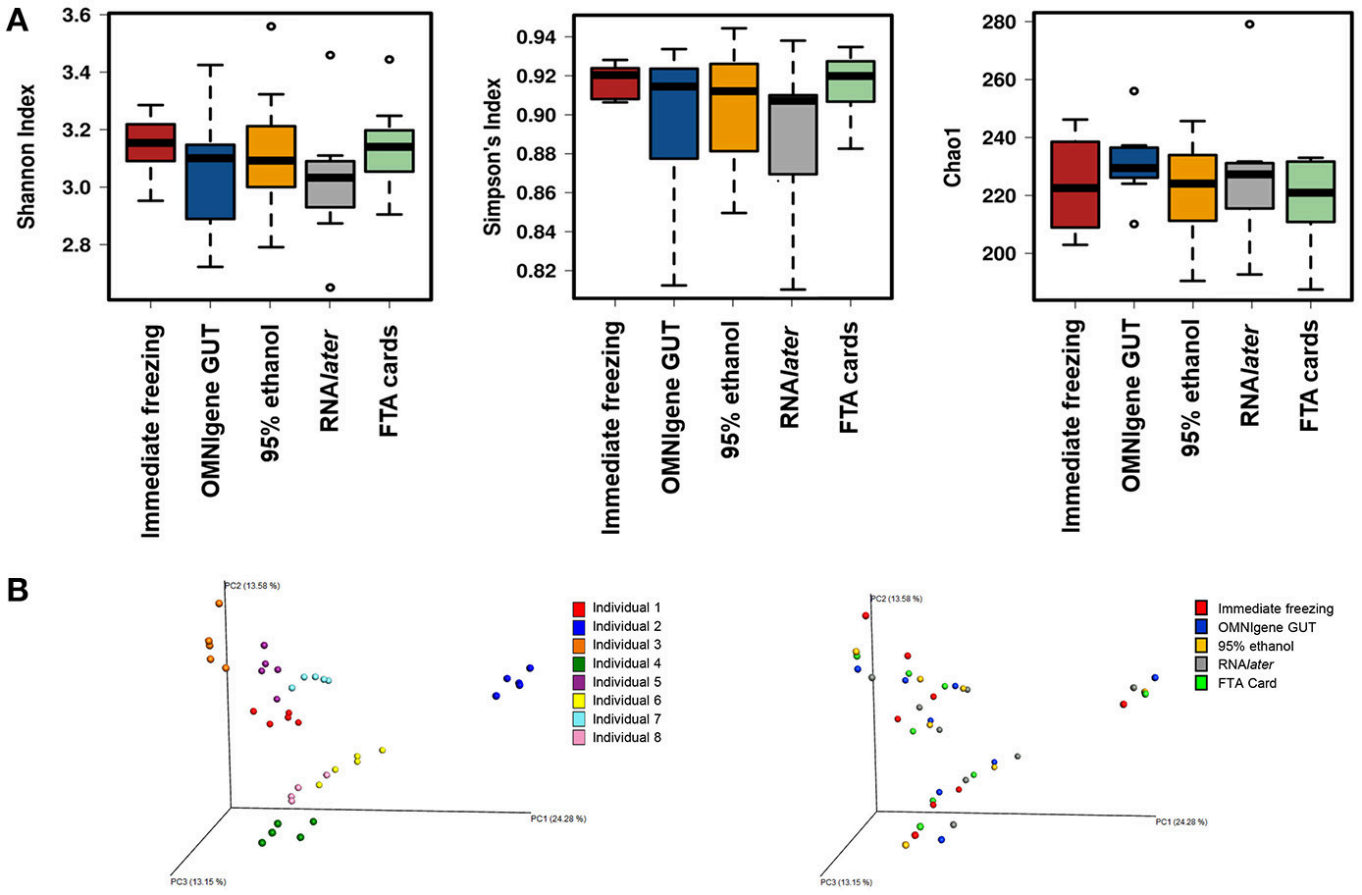
**(A)** α- diversity analyses for the five fecal collection methods. **(B)** Principal coordinates analyses (PCoAs) of β- diversity using Bray-Curtis distances.

**Figure 2:**
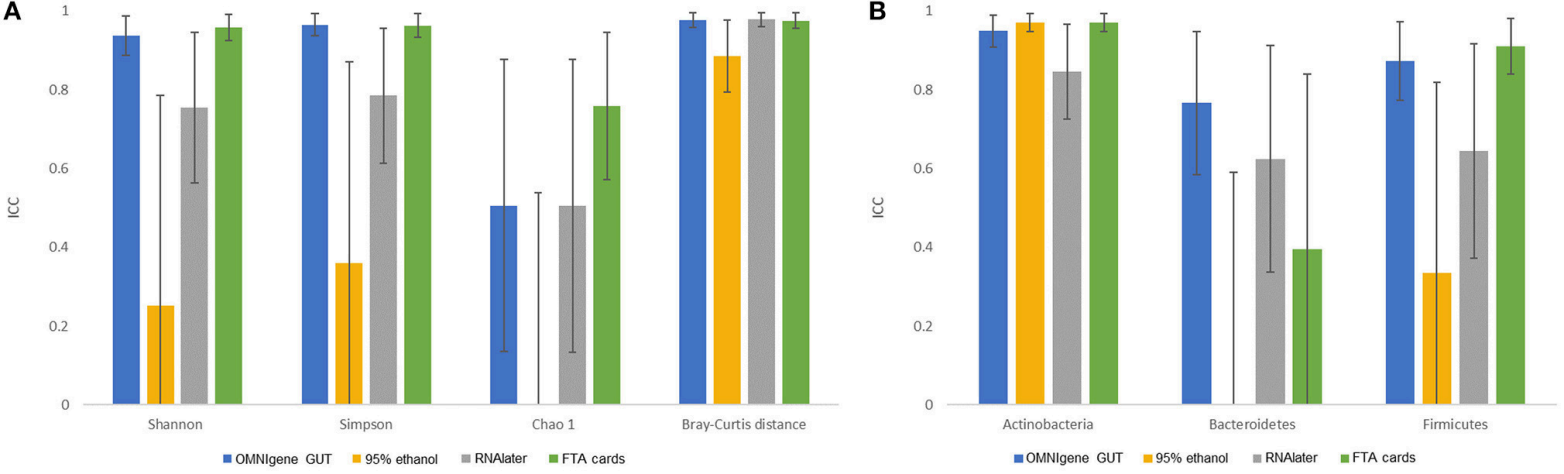
The concordance of microbiota obtained by different fecal collection methods compared with the gold standard immediate freezing: intraclass correlation coefficients (ICC) and 95% CI for: **(A)** three α- diversity metrics and β- diversity Bray-Curtis distance. **(B)** top three phyla.

There were no significant differences in α-diversity indices (Shannon index, Simpson's Index, Chao-1 Index) among the five fecal collection methods (all *P* > 0.05). The β- diversity Bray Curtis distance PCoA analysis indicated that inter-individual differences were responsible for the majority the microbial communities' variability, while very small differences were observed across different collection methods within samples (Figure 1). The FTA cards demonstrated the highest concordance with immediate freezing for three α-diversity indices (ICCs = 0.96, 0.96, 0.76 for Shannon index, Simpson's Index, Chao-1 Index, respectively), followed by the OMNIgene GUT (ICCs = 0.94, 0.97, 0.51), RNA*later* (ICCs = 0.75, 0.79, 0.51), and 95% ethanol (ICCs = 0.25, 0.36, 0.01) (Figure 2A). For the β-diversity metric, high ICCs (all >0.88) were observed for all four methods compared to immediate freezing.

Concordance results of the three pre-dominant phyla across collection methods are shown in Figure 2B. For Actinobacteria, the FTA cards (ICC = 0.97) and 95% ethanol (ICC = 0.97) showed the highest concordance with immediate freezing. OMNIgene GUT had nearly as high an ICC for Actinobacteria (0.95), while RNA*later* had a slightly lower ICC of 0.84. For Firmicutes, the FTA cards (ICC = 0.91) showed the highest concordance with immediate freezing, slightly exceeding the ICC for OMNIgene GUT (0.87), and substantially superior to RNA*later* (ICC = 0.64) and 95% ethanol (ICC = 0.34). For Bacteroidetes, OMNIgene GUT had the highest ICC although this only achieved a moderate concordance value of ICC = 0.76, where as the other samples showed relatively poor concordance with Immediate freezing. The taxonomic analysis at genus level across five collection methods is shown in Supplementary Figure S1.

### Metabolomics: untargeted metabolites

Using untargeted metabolomics, the number of metabolites detected in at least one fecal sample was 747 with immediate freezing, 694 with OMNIgene GUT, 742 with 95% ethanol, and 708 with FTA cards. Our attempts to measure metabolites in samples preserved with RNA*later* failed. We compared the four other methods at multiple levels, by further restricting to the subset of metabolites with ≥50, ≥75 and 100% detectability (Table 1). When we limited the data to the subset of metabolites that had ≥75% detectability, there were 621, 245, 467, and 376 metabolites in fecal samples collected with immediate freezing, OMNIgene GUT, 95% ethanol, and FTA cards, respectively. Compared with the gold standard immediate freezing which had 621 metabolites with 75% detectability in fecal samples, 95% ethanol showed the largest number of identical metabolites (*n* = 430; 69.2%). The next-best overlap of detected metabolites was observed for FTA cards (*n* = 330; 53.1%), followed by OMNIgene GUT (*n* = 231; 34.3%).

**Table 1:** Comparison of the number of metabolites at multiple detectability levels across different collection methods.

**Detectability level of metabolites**	**Methods**	**Total number of metabolites**	**Number of metabolites shared by GS and method (% of GS)**	**Number of known metabolites**	**Number of known metabolites shared by GS and method (% of GS known metabolites)**
All metabolites	Immediate freezing	747	–	131	–
	OMNIgene GUT	694	694 (92.9%)	128	128 (97.7%)
	95% ethanol	742	741 (99.2%)	132	131 (100%)
	FTA cards	708	707 (94.7%)	128	127 (97.0%)
≥50% detectability	Immediate freezing	705		129	–
	OMNIgene GUT	432	409 (58.0%)	106	105 (81.4%)
	95% ethanol	638	613 (87.0%)	126	124 (96.1%)
	FTA cards	518	494 (70.1%)	119	117 (90.7%)
≥75% detectability	Immediate freezing	621	–	123	–
	OMNIgene GUT	245	213 (34.3%)	74	72 (58.5%)
	95% ethanol	467	430 (69.2%)	118	112 (91.1%)
	FTA cards	376	330 (53.1%)	97	95 (77.2%)
100% detectability	Immediate freezing	393	–	104	–
	OMNIgene GUT	73	51 (13.0%)	29	27 (26.0%)
	95% ethanol	332	255 (65.9%)	97	88 (84.6%)
	FTA cards	192	140 (35.6%)	70	64 (61.5%)

*GS, gold standard*.

**Figure 3:**
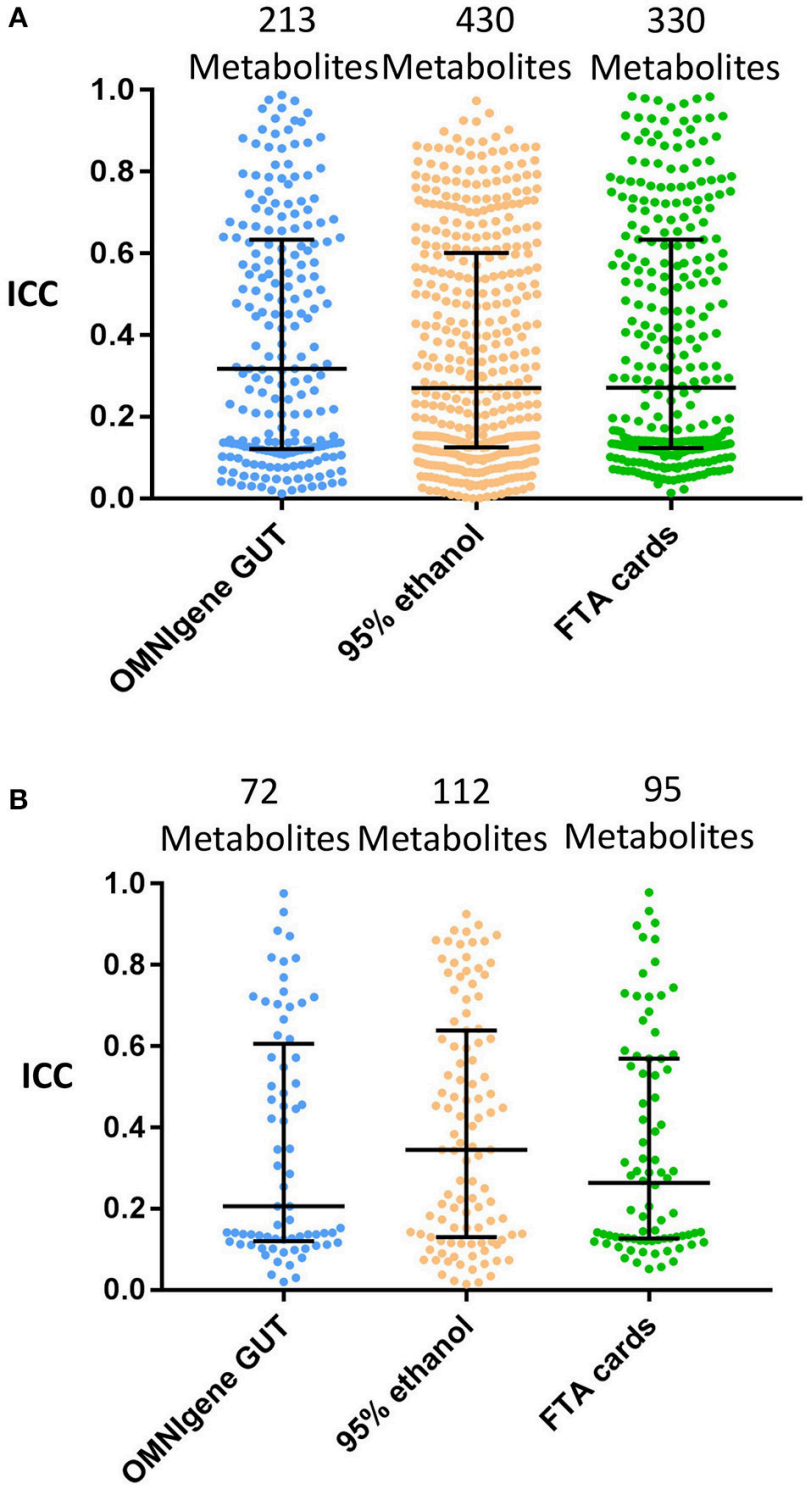
The concordance of metabolite detection by different fecal collection methods. Stool samples collected by the indicated methods were compared with the gold standard of immediate freezing for metabolite detection estimated the ICCs at ≥75% metabolites detectability level. Log10 transformation and Quantile normalization were used. Missing values were imputed with ½ minimum value for a given metabolite within one method. Highlighted medians and IQRs. **(A)** All Metabolites shared by GS and method. **(B)** Known (Named) Metabolites shared by GS and method.

The next concordance analyses were limited to the subset of metabolites with ≥75% detectability between the gold standard and each of the other collection methods (i.e., those metabolites above the ≥75% detection limit were included). The median of the ICCs (interquartile range; IQR) were 0.32 (0.12–0.63) for OMNIgene GUT, 0.27 (0.13–0.60) for 95% ethanol, and 0.27 (0.12–0.63) for FTA cards (Figure 3A).

Among annotated metabolites, the number detected in at least one fecal sample was 131 for immediate freezing, 128 for OMNIgene GUT, 132 for 95% ethanol, and 128 for FTA cards (Table 1). When the analysis was further restricted to the subset of annotated metabolites detected in ≥75% of samples, there were 123, 74, 118, and 97 metabolites in fecal samples collected with immediate freezing, OMNIgene GUT, 95% ethanol, and FTA cards, respectively (Table 1 and Supplementary Figure S2). Compared with immediate freezing which had 123 annotated metabolites with ≥75% detectability, 95% ethanol showed the largest number of identical metabolites (*n* = 112; 91.1%), followed by FTA cards (*n* = 95; 77.2%), and OMNIgene GUT (*n* = 72; 58.5%). Despite high to moderate concordance across methods in terms of the numbers of metabolites detected, the reproducibility (ICC) of measurements across methods was relatively poor. The median (IQR) of the ICCs for identical metabolites at 75% detectability level were 0.21 (0.12–0.61) for OMNIgene GUT, 0.35 (0.13–0.64) for 95% ethanol, and 0.26 (0.13–0.57) for FTA cards (Figure 3B).

### Metabolomic analyses: SCFAs

In total, 10 targeted SCFAs were detected in fecal samples collected with immediate freezing, OMNIgene GUT and FTA cards (Supplementary Table S1). SCFAs could not be detected in samples preserved in RNA*later*. All three collection methods had 100% detectability for the three predominant SCFAs: butyric acid, propionic acid, and acetic acid. For an additional set of seven measured SCFAs, detectability was 100% for immediate freezing, and detectability varied for OMNIgene GUT and FTA cards (Supplementary Table S1).

**Figure 4:**
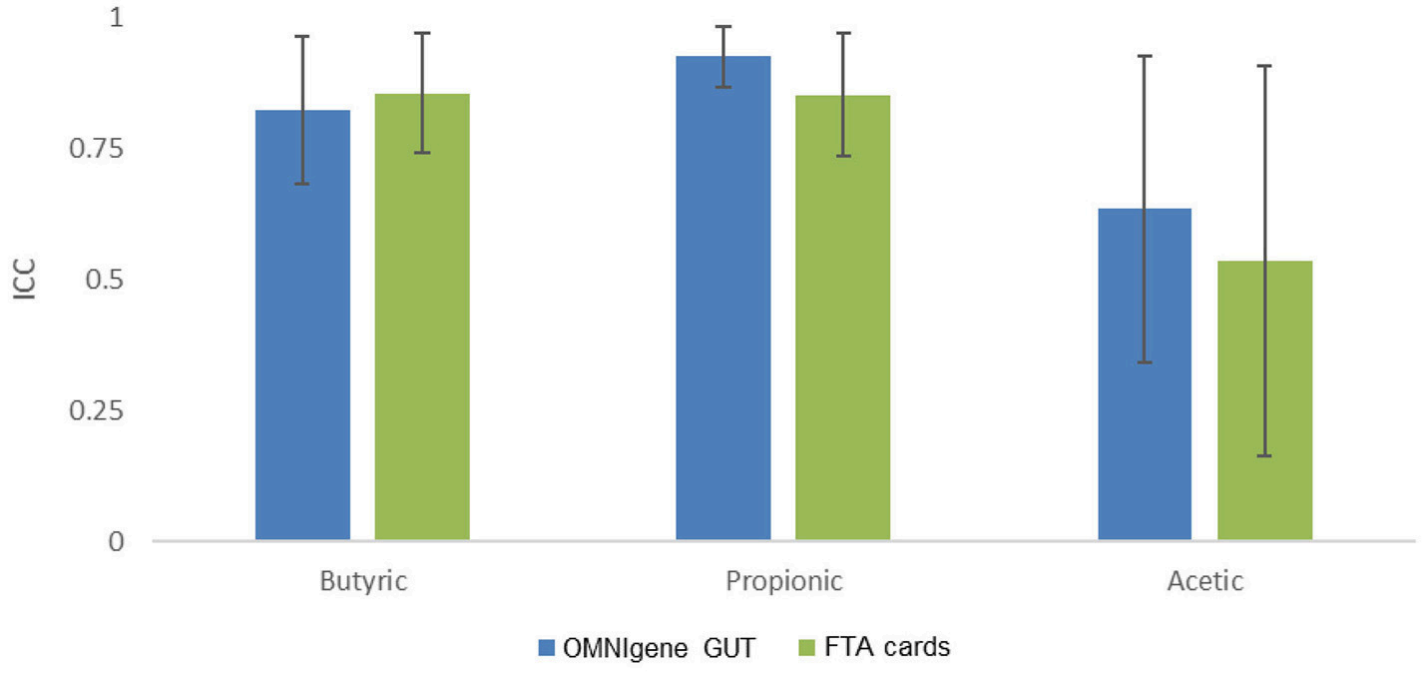
The concordance of fecal collection methods compared with the gold standard immediate freezing: intraclass correlation coefficients (ICC) and 95% CI for the three predominant SCFAs.

We then evaluated concordance of three predominant and seven other non-predominant SCFAs with ≥75% detectability between gold standard and the other methods (Figure 4). The ICCs for butyric acid were 0.82 (95% confidence interval [CI], 0.68 to 0.97) for OMNIgene GUT, 0.87 (95% CI, 0.74–0.97) for FTA cards. The ICCs for propionic acid were 0.93 (95% CI, 0.87–0.99) for OMNIgene GUT, and 0.85 (95% CI, 0.74–0.97) for FTA cards. The ICCs of Acetic acid were relatively lower for OMNIgene GUT (0.64, 95% CI, 0.34–0.93) and FTA cards (0.54, 95% CI, 0.16–0.91). For five non-predominant SCFAs, both methods showed very high concordance for isovaleric acid and hexanoate, but relatively lower concordance for isovaleric acid, valeric acid and 2-methylbutyric acid compared to immediate freezing (Supplementary Table S1).

### Bacterial populations vs. SCFA levels

We then explored the correlations between bacterial genera and the three predominant SCFAs in fecal samples. Nominally significant correlations between bacterial genera and SCFAs were observed. In immediate freezing fecal samples, we found nine correlations between bacterial genera and SCFAs (*P* < 0.05) (Table 2). The same trends for most correlations were observed in fecal samples collected with OMNIgene GUT and FTA cards, and some correlations were highly consistent across the three methods. For example, the correlation coefficients between *Blautia* and butyric acid were 0.83 (*P* = 0.01), 0.62 (*P* = 0.10), and 0.74 (*P* = 0.037) for immediate freezing, OMNIgene GUT and FTA cards, respectively. In addition, nominally significant correlations between the family *Rikenellaceae* and butyric acid, and between the genus *Faecalibacterium* and propionic acid were observed for FTA cards; and nominally significant correlations between the family *Clostridiales* and butyric acid, and between *Rikenellaceae* and butyric acid were observed for OMNIgene GUT.

**Table 2:** Short chain fatty acids associated taxonomy: Genera which were significantly correlated with butyric acid, propionic acid and acetic acid, by Spearman correlation coefficients.

	**Immediate freezing**		**OMNIgene GUT**		**FTA cards**	
	***r***	***P***	***r***	***P***	***r***	***P***
**BUTYRIC ACID**						
*Blautia*	0.83	0.010	0.62	0.102	0.74	0.037
*Rikenellaceae_UCG^#^*	−0.71	0.047	−0.91	0.002	−0.83	0.010
*Shuttleworthia*	−0.78	0.022	−0.59	0.123	0.07	0.867
*Bilophila*	−0.93	0.001^*^	−0.10	0.821	−0.57	0.145
*Clostridiales_UCG^#^*	−0.71	0.047	−0.91	0.002	−0.49	0.217
**PROPIONIC ACID**						
*Faecalibacterium*	0.88	0.004	0.45	0.260	0.74	0.037
*Eubacterium*	−0.76	0.029	−0.41	0.320	−0.31	0.453
**ACETIC ACID**						
*ML615J-28_UCG^#^*	0.75	0.033	0.17	0.694	0.38	0.359

* *P < 0.002 (critical P-value after Bonferroni correction)*.

# *UCG: unclassified genus. RNAlater samples could not be run for this assay*.

## Discussion

The immediate freezing without preservative has been widely used as a gold standard for gut microbiome analyses, as this method preserves microbial composition similar to analysis of a fresh sample and also avoids potential influence of added preservative (Flores et al., 2015; Loftfield et al., 2016; Song et al., 2016; Vogtmann et al., 2017b). In the current report, our data indicate that compared with the immediate freezing method, alternative specimen collection systems not requiring freezing including OMINIgene GUT, 95% ethanol, RNAlater, and FTA cards all can be informative for gut microbiome analysis (Song et al., 2016; Vogtmann et al., 2017b). All four methods showed relatively high concordance with the immediate freezing method for the β-diversity metric. Furthermore, there were small differences in microbial β-diversity Bray-Curtis distances across methods, while inter-individual differences were responsible for the majority of variation in microbial β-diversity. However, concordance for microbial α-diversity varied across different methods. The FTA cards and OMNIgene GUT showed relatively higher concordance with the immediate freezing for three α-diversity indices, followed by RNA*later* and 95% ethanol. Consistently, 95% ethanol was reported to have relatively low validity for α-diversity indices (Vogtmann et al., 2017b).

Interestingly, concordance for predominant microbial phyla varied across different methods as well as microbial phyla. For example, FTA cards had high ICCs (≥0.85) for Actinobateria and Firmicutes but not for Bacteroidetes (ICC = 0.40). Concordances of all four methods with the gold standard were generally high for the Actinobateria, but low for Bacteroidetes. Our results are supported by previous data (Vogtmann et al., 2017a), and previous studies that have indicated that some taxa are more sensitive to changes in collection and storage conditions (i.e., among immediate freezing, RNA*later* and TE buffer) (Choo et al., 2015). RNA*later* was reported to have decreased DNA purity which could interfere with downstream analyses (Dominianni et al., 2014). In addition, the cellular composition of Gram-positive and Gram-negative bacteria could be different, which presumably leads to differences in the ability to preserve DNA in some collection methods (Bahl et al., 2012; Fouhy et al., 2015). In our study, better concordances between methods were observed for the Actinobacteria and Firmicutes (both are Gram-positive) than that for Bacteroidetes (Gram-negative), which is consistent with a previous study (Vogtmann et al., 2017a). This may partially explain the low concordance for Bacteroidetes between methods, but the exact mechanisms remain unclear. Taken together, method-related variations should be carefully considered if multiple fecal collection methods are used in the analyses, though all four methods reported here are generally comparable to a frozen sample for gut microbiome analyses.

This study also compared different fecal sample collection methods for untargeted metabolomics. Our analyses at multiple detectability levels indicated 95% ethanol and FTA cards had comparable numbers of metabolites with high detectability when compared with immediate freezing. Particularly, among 132 known metabolites with ≥75% detectability in immediately frozen fecal samples, 91% and 77% of these metabolites were detected with 95% ethanol and FTA cards, respectively. However, OMNIgene GUT had many fewer detected metabolites. In a previous comparison study of untargeted metabolomics in fecal samples, 95% ethanol also showed the largest number and highest concordance of overlapping metabolites with the frozen sample, followed by FOBT cards (Loftfield et al., 2016). Given the existence of better alternative methods, OMNIgene GUT might not be recommended as the collection method for untargeted metabolomics in fecal samples. Another point worth noting is that RNAlater was not feasible for metabolomics measures. This was likely due to the high sodium sulfate content in the samples collected with RNAlater, which made the collection incompatible with mass spectrometry-based metabolomics platforms (Loftfield et al., 2016; Sinha et al., 2016).

An important contribution of this study is that we specifically compared fecal collection methods for targeted metabolomics on SCFAs, a family of disease-related metabolites highly relevant to gut microbiota. Our data indicated that for the most predominant SCFAs (butyric acid and propionic acid) both OMNIgene GUT and FTA cards had high concordance (all ICCs ≥0.80) with the immediate freezing method. For acetic acid, the ICCs were lower but still acceptable (0.64, 0.54). More importantly, we observed biologically plausible correlations between bacterial genera and the predominant SCFAs in fecal samples. Most correlations were reproduced with immediate freezing, OMNIgene GUT and FTA cards and the correlation coefficients were similar across these collection methods. For instance, we found that the genus *Blautia* was positively correlated with butyric acid using the frozen sample (*r* = 0.83) and this positive correlation was also observed by FTA cards (*r* = 0.74) and OMNIgene GUT (*r* = 0.62). This is in line with the fact that *Blautia* is a known butyrate producer (Berni Canani et al., 2016; Takahashi et al., 2016; Tanaka et al., 2016) and possesses the capability to produce butyric acid (Zhou et al., 2017). In addition, the negative correlation between *Rikenellaceae* and butyric acid was highly consistent across the frozen sample (*r* = −0.71), OMNIgene GUT (*r* = −0.94), and FTA cards (*r* = −0.83). Interestingly, this negative correlation has also been observed with rat fecal samples (Lin et al., 2016).

Besides a relatively small sample size, limitations of this study included the fact that participation was limited to healthy individuals. Future methodological work is need to ensure that results can be translated to other settings including patient groups and population-based epidemiological studies. Only one sample was collected for each method per individual in this study and we did not test reproducibility, although the reproducibility for these collection methods have been well-established. Finally, we did not examine the stability of each method over long-term storage. Many previous studies have reported relatively high stability of samples collected using FTA card or FOBT card, 95% ethanol and RNA*later* for microbial community analyses even preserved at ambient temperature for 4–14 days (Nechvatal et al., 2008; Franzosa et al., 2014; Flores et al., 2015; Voigt et al., 2015; Loftfield et al., 2016; Sinha et al., 2016; Song et al., 2016). Others have concluded that 95% ethanol and FOBT card collection methods were satisfactory for untargeted metabolomics analyses after 4 days, although not all methods tested in our study were evaluated in this prior study (Loftfield et al., 2016).

In summary, this study compared the concordance of the mainstream fecal collection technologies with the current gold standard method of immediate freezing for both microbiome and metabolomics analyses. The accuracy of FTA cards, OMNIgene GUT, and RNA*later* were acceptable for microbiome analyses, though variations, especially in taxonomy, were observed across different collection methods, similar to the findings in previous methodological studies (Flores et al., 2015; Song et al., 2016; Vogtmann et al., 2017a). For untargeted metabolomics, the concordance was highest with 95% ethanol and acceptable with FTA cards. Furthermore, this study, for the first time, indicated that FTA cards and OMNIgene GUT are generally comparable with the gold standard immediate freezing sample for measurement of SCFAs and integrated analysis of gut microbiota and SCFAs. Given the costs and technical challenges of collecting stool samples that are immediately frozen by participants and transported with cold packs, our data document comparable alternative methods for large epidemiological studies.

## Author contributions

ZW, RB and QQ analyzed data and wrote the manuscript. QQ, RB, RK, HS, and CI designed the experiments of this study. CZ, MU, and RB performed 16S rRNA gene sequencing experiments and ZW, MU, RB, and QQ analyzed gut microbiota data. YQ and IK performed untargeted and targeted metabolomics experiments and ZW, QQ, YP, TW, and IK analyzed the metabolomics data.

### Conflict of interest statement

The authors declare that the research was conducted in the absence of any commercial or financial relationships that could be construed as a potential conflict of interest.
