# Supplementary material for: Comparison of Fecal Collection Methods for Microbiome and Metabolomics Studies
Source: Front Cell Infect Microbiol. 2018 Aug 28;8:301. doi: 10.3389/fcimb.2018.00301 (PMC6127643; doi:10.3389/fcimb.2018.00301)

Table S1． Comparison of the detectability of short-chain fatty acids in all samples.

SCFAs	Immediate freezing	OMNIgene GUT		FTA cards	
	Detectability	Detectability	ICCs	Detectability	ICCs
Butyric acid	100.0%	100.0%	0.82	100.0%	0.86
Propionic acid	100.0%	100.0%	0.93	100.0%	0.85
Acetic acid	100.0%	100.0%	0.64	100.0%	0.54
Valeric acid	100.0%	87.5%	0.63	75.0%	0.47
Isovaleric acid	100.0%	100.0%	0.78	87.5%	0.19
Hexanoate	100.0%	100.0%	0.98	100.0%	0.96
2-Methylbutyric acid	100.0%	100.0%	0.80	87.5%	0.17
Isobutyric acid	100.0%	100.0%	0.94	100.0%	0.97
4-Methylvaleric acid	100.0%	87.5%	NA	25.0%	NA
3-Methylpentanoic acid	100.0%	50.0%	NA	12.5%	NA

RNA*later* not shown since unable to analyze.

Figure S1. Taxonomic analysis at the genus level for each fecal sample across all collection methods.

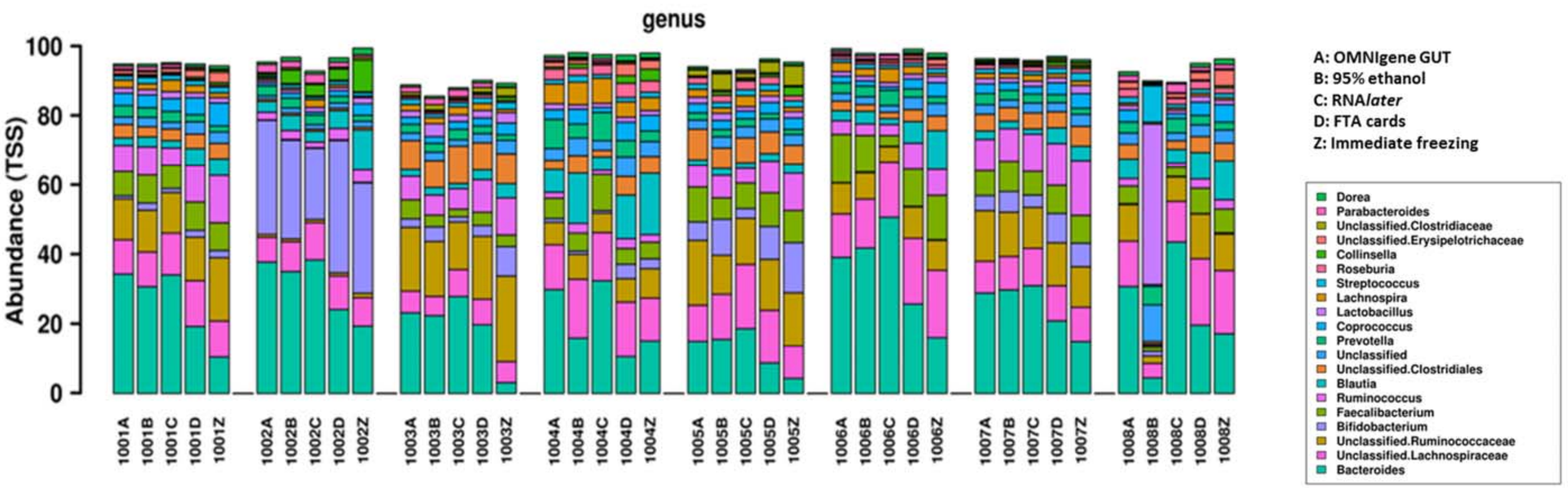

Figure S2. Number of metabolites in fecal samples by collection method and metabolites categorized at the "≥75% detectability" level and "all metabolites" level

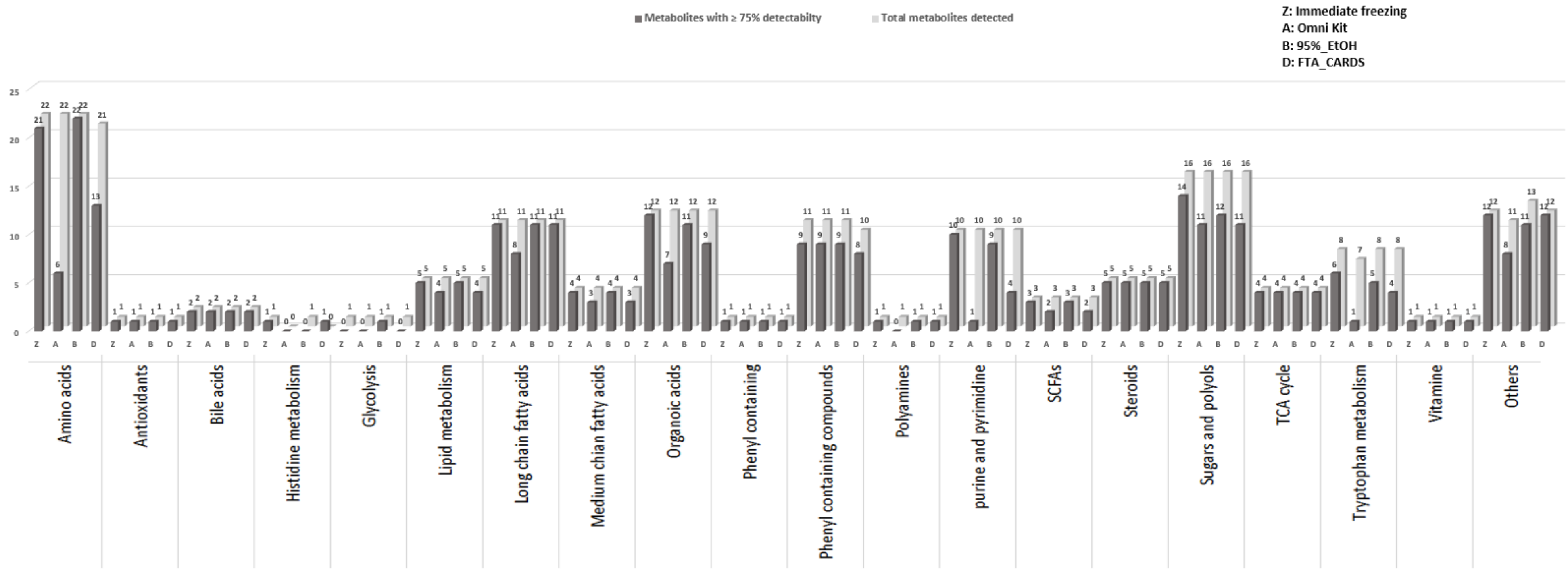

Supplement: Supplementary file 1 [file Data_Sheet_1.PDF]
